# Arthritis Caused by *Nannizziopsis obscura*, France

**DOI:** 10.3201/eid2809.220375

**Published:** 2022-09

**Authors:** Hélène Mascitti, Valérie Sivadon-Tardy, Marie-Elisabeth Bougnoux, Clara Duran, Mickael Tordjman, Marie-Alice Colombier, Isabelle Bourgault-Villada, Aurélien Dinh

**Affiliations:** Raymond-Poincaré University Hospital, Public Assistance–Paris Hospitals, Paris Saclay University, Garches, France (H. Mascitti, C. Duran, A. Dinh);; Ambroise-Paré University Hospital, Public Assistance–Paris Hospitals, Paris Saclay University, Boulogne-Billancourt, France (V. Sivadon-Tardy, M. Tordjman, I. Bourgault-Villada);; Necker University Hospital, Public Assistance–Hospitals of Paris, University of Paris, Paris, France (M.-E. Bougnoux);; Foch Hospital, Suresnes, France (M.-A. Colombier)

**Keywords:** Arthritis, *Nannizziopsis obscura*, fungi, septic arthritis, necrotizing cellulitis, sepsis, France

## Abstract

*Nannizziopsis* spp., fungi responsible for emerging diseases, are rarely involved in human bone and joint infections. We present a rare case of septic arthritis with necrotizing cellulitis caused by *N. obscura* in a patient in France who had undergone kidney transplant. Rapid, aggressive medical and surgical management led to a favorable outcome.

*Nannizziopsis* spp. are keratinophilic fungi that can cause aggressive pyogranulomatous lesions affecting the skin, integument, and musculoskeletal systems of reptiles. The ecology of these fungi is not well known, and human infections are rarely. We report a case of septic arthritis caused by *N. obscura.*

In April 2019, a 56-year-old man, originally from Senegal and a former taxi driver in France, was hospitalized in the intensive care unit of Ambroise-Paré Hospital, Boulogne-Billancourt, France, for renal failure and sepsis. He had a prior diagnosis of diabetes mellitus, had undergone kidney transplant 6 years earlier for interstitial kidney disease, and had been undergoing dialysis during the prior year. His usual medication regimen included tacrolimus (4 mg 2×/d), prednisolone (5 mg 2×/d), and mycophenolate mofetil (250 mg 4×/d; stopped at admission).

At admission, the patient was hypothermic (34.9°C) and had moderate impaired consciousness. Blood pressure was 152/85 mm Hg and heart rate 95 bpm. No septic shock was observed. The patient’s right leg showed swelling, redness, and tenderness. Testing revealed creatinine level of 333 µmol/L, leukocyte count of 5.6 G/L, neutrophil count of 4.77 G/L, and creatine phosphokinase level of 26 IU/L. A computed tomography scan of the right lower limb revealed ankle joint effusion associated with gas, compatible with septic arthritis (Figure). On the basis of these findings and the observance of concomitant necrotizing cellulitis, the patient underwent immediate surgery, which involved debridement of the skin and subcutaneous tissues of the dorsal face of the right foot and the medial face of the distal tibia, including excision of necrotic tissue (fascia and muscle). Abundant purulent discharge was observed during surgery.

**Figure:**
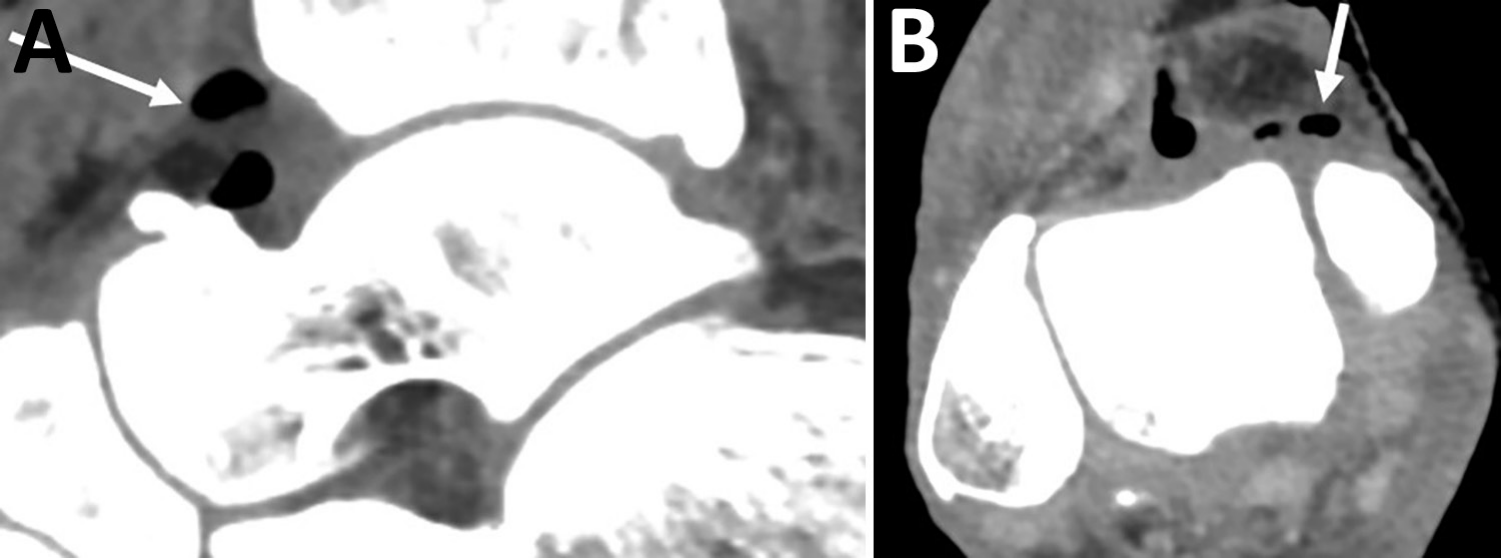
Sagittal (left) and axial (right) contrast-enhanced computed tomography images demonstrating ankle joint effusion associated with gas (arrows) in an immunocompromised man in France who had septic arthritis and soft tissue infection caused by *Nannizziopsis obscura.*

Because direct histologic examination of intra-operative samples revealed several septate and arthrosporous mycelial filaments, we prescribed empiric treatment that included liposomal amphotericin B and broad-spectrum antibiotics. Because no bacteria were isolated, we stopped antibiotic therapy 5 days after surgery. The patient underwent additional surgeries 48 and 96 hours after initial surgery based on a diminishing clinical course and persistence of the previously observed fluid collections and air pockets on computed tomography scan, revealing effusion of the right tibio-talar joint and arthritis.

A subculture on Sabouraud dextrose agar with chloramphenicol yielded white, cottony colonies after 3–5 days. Using molecular identification by PCR amplification and sequencing of internal transcribed spacer (ITS) regions (ITS1–5,8S -ITS2), the clinical isolate was identified as *N. obscura,* confirmed by matrix-assisted laser desorption/ionization time-of-flight mass spectrometry. An antifongigram revealed a strain sensitive to azole antifungals (itraconazole [MIC 0.380 µg/mL], isavuconazole [MIC 0.064 µg/mL], voriconazole [MIC 0.008 µg/mL], and posaconazole [MIC 0.190 µg/mL]), micafungin (MIC 0.023 µg/mL), and amphotericin B (MIC 0.125 µg/mL). Serum β-D-glucan level was elevated (>500 pg/mL).

Because the *Nannizziopsis* strain was sensitive to voriconazole, we prescribed an initial oral regimen of the drug (3 mg/kg 2×/d) on day 6 of the initial intervention, without a loading dose. Drug monitoring revealed a plasma drug concentration of 0.7 mg/L, which was below the therapeutic threshold (1–2 mg/L), due to an ultra-rapid metabolizer profile after the genotyping of CYP2C19*17/*17. Voriconazole was then stopped and replaced by a regimen of posaconazole (loading dose, 300 mg 2×/d; maintenance dose, 300 mg/d) for a 1-year period. The patient’s plasma drug concentration was 0.6 mg/L, above the therapeutic threshold (0.5 mg/L).

Computed tomography scans of the head, chest, abdomen, and pelvis and transesophageal cardiac ultrasound performed 2 months after admission revealed no lesion. The patient resumed immunosuppressive therapy with prednisolone (initiated after his kidney transplant) within 30 days of the last surgery for this soft-tissue infection. Tacrolimus, which was initiated at a reduced dosage (1.5 mg 2×/d) earlier in the patient’s course of treatment, was then stopped. One year after his septic episode, the patient had no recurrence and had a partially functional lower right limb.

Only 14 cases of invasive *Nannizziopsis* infections in humans have been reported (*1*–*5*). These infections can be acute or chronic and usually occur in immunocompromised patients. Human infections caused by *Nannizziopsis* are generally cutaneous and subcutaneous, but some cases of pulmonary infection have been noted (*6*,*7*). Probabilistic treatment with azoles is recommended in the absence of a definitive diagnosis. Because serum β-D-glucans are typically very high during infections with *Nannizziopsis* spp., detection of a very high serum β-D-glucan level may guide the diagnosis of invasive fungal infection (*8*).

Given the wide variations in patient response and the potential impact of drug interactions, antifungal treatment should include serum drug monitoring to guide drug selection and optimize treatment dosage. Prognosis still largely depends on the underlying immunosuppression of the patient and the outcome of surgical management. This patient reported no recent travel to Africa, no history of cutaneous lesion or infection, and no recent contact with reptiles, which suggests that severe *Nannizziopsis* infection could occur several years after possible exposure among immunosuppressed patients.

This rare case of septic arthritis due to *N. obscura* occurred secondarily to a skin and soft tissue infection. Such severe infections require urgent medico-surgical treatment; the probability of a favorable outcome often diminishes when diagnosis and treatment are delayed. Medical treatment for infections caused by *N. obscura* is based on antifungals from the azole class, and dosages must be carefully monitored. 
